# Aromatic Hyperbranched Polyester/RTM6 Epoxy Resin for EXTREME Dynamic Loading Aeronautical Applications

**DOI:** 10.3390/nano10020188

**Published:** 2020-01-22

**Authors:** Aldobenedetto Zotti, Ahmed Elmahdy, Simona Zuppolini, Anna Borriello, Patricia Verleysen, Mauro Zarrelli

**Affiliations:** 1 Institute for Polymers, Composites and Biomaterials, National Research Council of Italy, P.le Fermi, 1, 80055 Portici, Italy; aldobenedetto.zotti@unina.it (A.Z.); simona.zuppolini@cnr.it (S.Z.); mauro.zarrelli@cnr.it (M.Z.); 2 Department of Electromechanical, Systems & Metal Engineering, MST-DyMaLab, Ghent University, Technologiepark 46, B-9052 Zwijnaarde, Belgium; ahmed.elmahdy@UGent.be

**Keywords:** thermosetting resin, polymer-matrix composites (PMCs), fracture toughness, high-strain rate mechanical properties, hyperbranched polymers (HBPs)

## Abstract

The effects of the addition of an aromatic hyperbranched polyester (AHBP) on thermal, mechanical, and fracture toughness properties of a thermosetting resin system were investigated. AHBP filler, synthesized by using a bulk poly-condensation reaction, reveals a glassy state at room temperature. Indeed, according to differential scanning calorimetry measurements, the glass transition temperature (T_g_) of AHBP is 95 °C. Three different adduct weight percentages were employed to manufacture the AHBP/epoxy samples, respectively, 0.1, 1, and 5 wt%. Dynamical Mechanical Analysis tests revealed that the addition of AHBP induces a negligible variation in terms of conservative modulus, whereas a slight T_g_ reduction of about 4 °C was observed at 5 wt% of filler content. Fracture toughness results showed an improvement of both critical stress intensity factor (+18%) and critical strain energy release rate (+83%) by adding 5 wt% of AHBP compared to the neat epoxy matrix. Static and dynamic compression tests covering strain rates ranging from 0.0008 to 1000 s^−1^ revealed a pronounced strain rate sensitivity for all AHBP/epoxy systems. The AHBP composites all showed an increase of the true peak yield compressive strength with the best improvement associated with the sample with 0.1 wt% of AHBP.

## 1. Introduction

Epoxy resins are widely used in different application fields such as aerospace [1] (matrix in composites), electronics [2] (high-performance protective coatings), and structures [3,4] (anti-corrosive coatings and structural adhesives) because of their low specific weight compared to metals, good mechanical properties, and high corrosion resistance.

Epoxy resins are brittle in nature [5], and their poor impact resistance limits their use in advanced applications for which a high toughness is required, such as adhesives and fiber-reinforced polymers for aerospace [6,7]. In order to enhance their fracture toughness, fillers with different typologies have been employed, such as rubber particles [8], inorganic nanoparticles [9], carbon nanotubes [10], and hyperbranched polymers [11,12]. All these filler categories have the potential to increase the toughness of epoxy resins, though they might compromise the strength to varying degrees.

Hyperbranched polymers (HBPs) have been explored as novel, high potential, low viscosity tougheners for epoxy resins, as they increase fracture toughness properties without remarkable detrimental effects on other properties. Their non-branched counterparts can drastically reduce the T_g_ of the resin, deteriorate the thermal stability and storage modulus, and unavoidably limit the processability due to the significant increase of the matrix viscosity [13]. HBPs, on the contrary, are known to exhibit lower melt and solution viscosities compared to linear polymers of the same molar mass, mainly due to their unique structure [12]. Another important advantage of HBPs is that their simplified synthetic procedure enables production in large quantities, thus making their use in engineering applications potentially economically viable.

Fu et al. [14] have toughened a diglycidyl ether of bisphenol A (DGEBA) resin system using a hyperbranched polyester (poly(trimellitic anhydride-triethylene glycol) ester epoxy-HTTE). Their study focused on the effect of the hyperbranched generation number on the thermo-mechanical properties and impact resistance of the epoxy system. The authors found an improvement of the impact strength with a factor of 2 to 7 for HTTE/DGEBA blends compared to the unmodified system. The addition of the HBP reduces the T_g_ of the epoxy system. However, the reduction of T_g_ decreases with the increase of the HBP generation number. Similar behavior is observed for the thermal stability of the nanocomposite. In some cases, HBPs are able to increase the fracture toughness and impact resistance of the hosting matrix without loss of thermo-mechanical properties, as demonstrated by Boogh et al. [15]. In their work, the addition of 5 wt% of HBP results in an increase of the critical strain energy release rate (*G_IC_*) from 120 J/m^2^ for the neat resin to 720 J/m^2^ for the modified one. The HBP addition does not lead to a decrease of both resin elastic modulus and T_g_, and there are not remarkable changes in resin viscosity. As such, the epoxy processability is fully maintained without the additional use of solvents. The absence of a remarkable loss in thermo-mechanical properties could be attributable to the strong mechanical interaction between epoxy matrix and the reactive epoxy groups grafted onto the HBP. Xu et al. [16] reported an increase in curing rate for a DGEBA-based epoxy system loaded with hyperbranched polyester with hydroxyl end groups (HBP-OH). The faster curing process is ascribable to the catalytic effect of hydroxyl groups in HBP-OH and the low viscosity of the blend at curing temperature. In addition, the fracture toughness of the cured system with 20 wt% of HBP-OH has been studied. The critical stress intensity factor (*K_IC_*) value is about twice the value of pure epoxy resin. HBPs are also employed as shell in core/shell nanoparticles. An example is reported by Wei et al. [17], who used an aromatic hyperbranched polyamide as shell for magnetic Fe_3_O_4_ nanoparticles in order to improve the dispersion within the hosting matrix. The choice of using an HBP as a shell is motivated by the higher surface functionality compared to their linear polymer counterpart. This feature is extensively used in applications where a larger interaction between filler and matrix is needed in order to improve the stress transfer [18] or the dispersion [17,19].

As mentioned above, one of the aims of toughening epoxy resins is to increase their fracture toughness and impact resistance. This is necessary for the use of epoxy resins in various aeronautical components. Since these components are typically subjected to impact loads, it is essential to study the high strain rate behavior of epoxy resins. Several researchers have studied the high strain rate tensile and compressive behaviors of epoxy resins. Gerlach et al. [20] have investigated the tensile and the compressive behaviors of RTM6 epoxy at strain rates up to 10^4^ s^−1^. Results showed that the RTM6 epoxy resin is strain rate sensitive in both tension and compression. Both yield strength and initial modulus increased with increasing strain rates. Rio et al. [21] and Naik et al. [22] also reported an increase of the elastic modulus and the compressive yield strength for epoxy resins with increasing strain rates up to 2500 s^−1^. Gilat et al. [23] have studied the high strain rate behavior of untoughened E-862 and toughened PR-520 epoxy resins in tension and shear up to strain rates of 700 s^−1^. Results also indicated that both epoxy resins are strain rate sensitive in tension and shear. The toughened resin showed higher tension and shear strengths compared to the un-toughened resin at high strain rates. Guo et al. [24] have studied the compressive response of a silica/epoxy system at high strain rates up to 10^4^ s^−1^. The authors demonstrated that the influence of the nanoparticle on the mechanical properties of the epoxy matrix are strongly affected by both strain rate and dispersion of the nanoparticles. Indeed, the silica/epoxy systems exhibit a better performance when the dispersion is uniform, i.e., without filler clusters. Moreover, the compression stress goes up dramatically as the strain-rate increases. Shadlou et al. [25] have investigated the effect of strain rate on the mechanical behavior of epoxy reinforced with graphene nanoplatelets. Results have shown that the compressive modulus and strength of nanocomposites increase with increasing strain rates. Moreover, it was demonstrated that the effect of the nanoplatelets reinforcement decreases at higher strain rates.

Despite the availability of several studies on the dynamic response of neat and toughened epoxies by different toughening particles, very few studies are available on the dynamic behavior of hyperbranched polymer toughened epoxy. Li et al. [26] have studied the effect of hyperbranched core-shell nanoparticles on the tensile and impact resistance of epoxy resin. Results showed that the addition of 3% hyperbranched particles can significantly improve the tensile and impact resistance of the epoxy resin.

Although hyperbranched polymers have a proven potential as toughening agent for epoxy resins, they are generally characterized by a low T_g_. This, in turn, could eventually reduce the T_g_ of the hosting matrix [13]. One possible solution to this issue is to use a hyperbranched polymer with a high T_g_, in order to limit the reduction of the T_g_ of the hosting epoxy matrix. This could have a significant effect on the mechanical performance of the resulting epoxy blend in general, and on the high strain rate mechanical performance in particular. Therefore, the aim of this work is to study the thermo-mechanical properties and the fracture toughness performance of an aeronautical grade epoxy resin modified with a hyperbranched polyester characterized by a high T_g_. Additionally, the compressive behavior of the synthesized aromatic hyperbranched polyester (AHBP)/epoxy nanocomposites is investigated at static and high strain rates using a conventional test machine and split Hopkinson pressure bar setup, respectively.

## 2. Materials and Methods

### 2.1. Experimental

**Figure 1:**
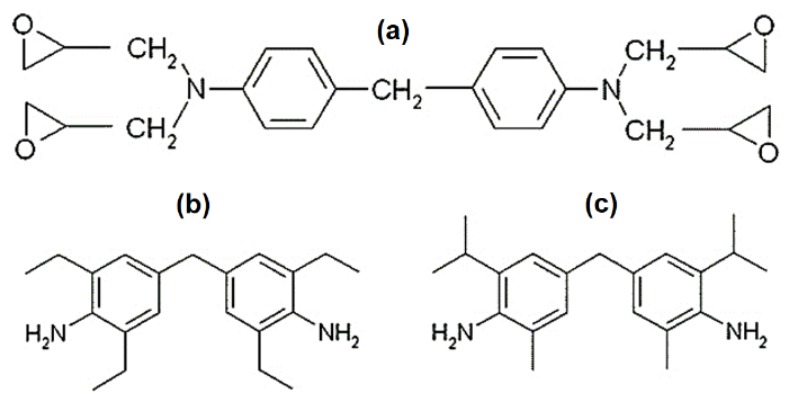
Chemical structure of (**a**) tetraglycidyl methylene dianiline, (**b**) 4,4′-methylenebis(2,6-diethylaniline), and (**c**) 4,4′-methylenebis(2-isopropyl-6-methylaniline) [16].

The epoxy matrix, RTM6, was supplied by Hexcel Composites (Duxford, UK). RTM6 is a premixed epoxy-amine system, composed by the tetra-functional epoxy resin tetraglycidyl methylene dianiline (TGMDA, Figure 1a) and two hardeners: 4,4′-methylenebis(2,6-diethylaniline) and 4,4′-methylenebis(2-isopropyl-6-methylaniline) (Figure 1b) [27]. RTM6 is developed for the resin transfer molding process, and is characterized by an epoxy equivalent weight of 116 g/eq [28] and a minimum viscosity of 33 mPa·s at 120 °C [29].

The 4,4-bis(p-hydroxyphenyl)pentanoic acid (diphenolic acid), tin(II) 2-ethylhexanoate (92.5–100%), Sn(Oct)_2_, and tetrahydrofuran (99.9%, THF) were purchased by Sigma-Aldrich and used as received.

### 2.2. Synthesis of AHBP

**Figure 2:**
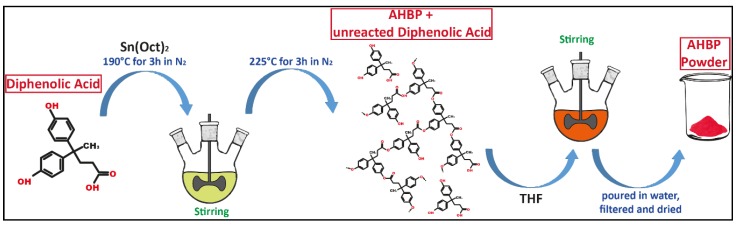
The synthesis procedure of aromatic hyperbranched polyester (AHBP).

AHBP synthesis was performed according to a modified procedure reported in literature [30]. Diphenolic acid (5.0 g) and Sn(Oct)_2_ (60 μL) were magnetic stirred in a three-necked round-bottom flask at 190 °C under nitrogen atmosphere for 3 h, to ensure the homogeneity of the mixture. Then, the temperature was raised up to 225 °C to trigger the polymerization and the reaction was kept stirring for 3 h. The crude product was dissolved in 10 mL of THF, and then the solution was poured in a large amount of deionized water (≈0.5 L) in order to precipitate the AHBP. Subsequently, the precipitate was filtered, washed with deionized water, and then dried in an oven under vacuum at 100 °C, for a night. The obtained AHBP was characterized by a red/brown color. Figure 2 shows a schematization of the synthesis procedure.

### 2.3. Preparation of Epoxy Thermoset

To prepare the epoxy/AHBP nanocomposites, an appropriate amount of AHBP was dissolved in 5 mL of THF and the solution was mixed with the epoxy resin in a round-bottom flask. The solvent was removed by a rotavapor at 90 °C. The resulting mixture was poured in a stainless steel template, previously coated with a release agent (FREKOTE 70), and then cured at 160 °C, for 90 min, followed by a post-curing stage of 2 h at 180 °C. The cured plates were cooled down slowly in the oven overnight to room temperature, removed from the template, and cut to the prescribed sample dimensions for the programmed mechanical tests.

Three different filler concentrations were employed: 0.1, 1, and 5 wt%.

### 2.4. Characterization

The molecular weight and its distribution were determined by a Waters Gel Permeation Chromatography (GPC, Waters Associates, Milford, MA, USA) 515–2410 system with THF as the eluent.

Nuclear magnetic resonance (NMR) spectra were recorded on a Brucker Advance 400 MHz spectrometer (Milano, Italy) operating at 400 and 100 MHz for ^1^H and ^13^C, respectively. DMSO-d_6_ was used as solvent and chemical shifts (δ) are reported in ppm relatively to the residual solvent signal (DMSO-d_6_: δ_H_ 2.54, δ_C_ 39.7). For ^13^C-NMR a standard pulse sequence zgpg30 (Bruker library) was used and 1 s recycle delay, 1.2 s acquisition time, and a pulse angle of 30° were applied, attaining conditions close to the maximum signal-to-noise ratio and far from any saturation. Spectra were processed by Top Spin 3.1 software (Bruker).

Fourier transform infrared (FTIR) spectra were recorded on a Perkin Elmer Spectrum 100 FTIR spectrophotometer (Milano, Italy) in the 4000–400 cm^−1^ region, with a resolution of 1 cm^−1^, using attenuated total internal reflectance spectroscopy (ATR).

The rheological characterization of the AHBP/RTM6 suspension was carried out by a MRC-301 rheometer (Anton Paar, Graz, Austria), using parallel plates PP25 with a diameter of 25 mm with a 0.5 mm gap. Temperature ramp tests were performed in order to study the viscosity changes of the AHBP/RTM6 suspensions with temperature. The heating rate was set to 2 °C/min from ambient temperature to the temperature associated with a viscosity value equal to 10^6^ Pa·s. Frequency and deformation were fixed, i.e., 1 Hz and 0.5%, respectively.

T_g_ of synthesized hyperbranched and manufactured nanocomposites were evaluated using a Differential Scanning Calorimeter (DSC) Q1000 by TA Instruments (New Castle, Germany), under a nitrogen atmosphere (50 mL/min) and a heating ramp of 10 °C/min. The samples used had a weight of 5 ± 0.5 mg.

Dynamic mechanical analysis (DMA) was performed using the Q800 DMA by TA Instruments at a fixed frequency of 1 Hz and heating ramp of 3 °C/min. The testing configuration was the double cantilever mode with nominal sample dimensions of 60 × 10 × 2.5 mm^3^. An amplitude of 60 μm was considered for all the tests.

Mode-I fracture tests were conducted using Single Edge Notched Beam (SENB) specimen. The test specimen was chosen according to the ASTM D5045-99 standard test method. To ensure the plane strain conditions, samples with nominal dimensions of 3 × 6 × 27 mm^3^ were employed. According to the ASTM standard, the crack length should be selected such that 0.45 < a/W < 0.55. The crack was obtained in two steps: first a sharp crack was machined with a saw, then a natural crack was initiated by sliding a new razor blade across the notch root. The fracture tests were performed using an Instron 4301 equipped with a 250 N load cell at a displacement rate of 1 mm/min.

*K_IC_* values were calculated according to the standard using the following equation:

(1) $$K_{IC}=f(a/w)\left(\frac{P_{Q}}{BW^{1/2}}\right),$$

(2) $$f\left(\frac{a}{w}\right)=6\left(\frac{a}{w}\right)\frac{\left[1.99-\left(\frac{a}{w}\right)\left(1-\frac{a}{w}\right)\left(2.15-3.93\frac{a}{w}+2.7{\left(\frac{a}{w}\right)}^{2}\right)\right]}{\left(1+2\frac{a}{w}\right){\left(1-\frac{a}{w}\right)}^{3/2}},$$

where *P_Q_* is the load at failure, a crack length, while *W* and *B* are the specimen width and thickness, respectively. *G_IC_* were calculated from the stress intensity values according to the cited ASTM standard. Scanning electron microscopy (SEM) observations (FEI Quanta 200 FEG, ThermoFisher Scientific, Hillsboro, OR, USA) were performed on the nanocomposites fractured surfaces obtained as a result of fracture toughness tests, in order study the fracture mechanisms involved during the nanocomposites breakage.

**Figure 3:**
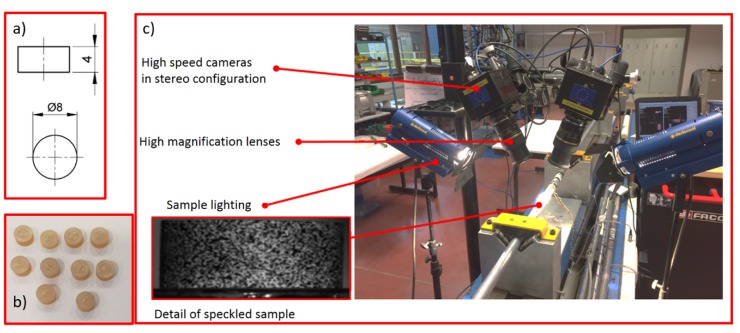
(**a**) Dynamic compression test sample geometry, (**b**) picture of the manufactured samples, and (**c**) detail of the split Hopkinson pressure bar setup used for the dynamic compression tests.

High strain rate compression experiments were carried out using the split Hopkinson compression bar setup of DyMaLab at Ghent University. Cylindrical samples of neat RTM6 and modified RTM6 resin with three AHBP concentrations (i.e., 0.1, 1, and 5 wt%) were manufactured for these experiments. The diameter and the height of the cylindrical samples were 8 and 4 mm, respectively. The setup consisted of two aluminum bars, called input and output bars, each having a diameter of 25 mm. Both bars were carefully aligned and allowed to move freely on horizontal supports. The length of the input bar was 6 m while the length of the output bar was 3.25 m. Samples were positioned and aligned between the two bars. During a high-speed compression experiment, a compressive incident wave is generated by accelerating an impactor towards the free end of the input bar. This compressive incident wave propagates along the input bar towards the sample. The incident wave is partly reflected and partly transmitted by the sample. The strain histories corresponding to the incident, reflected, and transmitted waves were measured by means of strain gauges attached at well-chosen points on the input and output bars. Using the theory presented by Kolsky [31], the time histories of the force, the global deformation, and velocity imposed to the sample can be derived from the measured waves. The amplitude of the incident wave is proportional to the velocity of the impactor and can be tuned: a higher impactor velocity results in a higher strain rate in the sample. For each material, tests were performed with impactor velocities of 8, 11, and 14 m/s. Additionally, the digital image correlation (DIC) technique was used to obtain full-field strain measurements locally on the surface of the samples. To this purpose, two high-speed cameras positioned under a certain angle were used to record the deformation of a speckle pattern applied to the sample surface prior to testing. Figure 3 shows the dynamic compression setup used.

**Figure 4:**
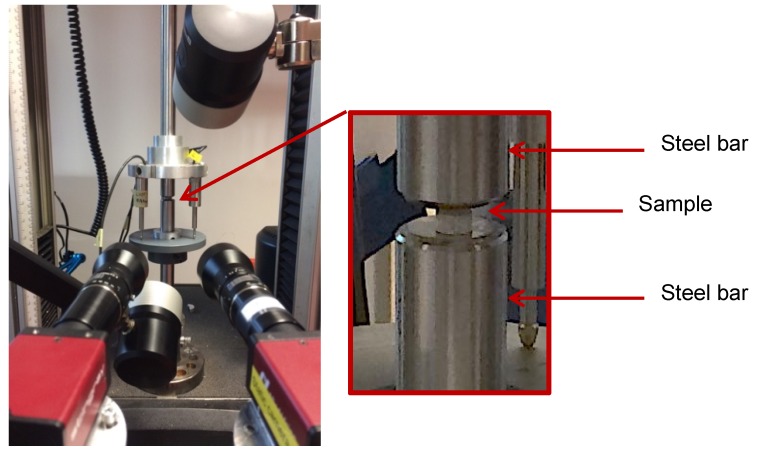
Quasi-static setup used with digital image correlation (DIC) and displacement transducers with detail on sample positioning.

Next to the dynamic compression tests, quasi-static reference tests were performed on an Instron 5569 universal testing machine (see Figure 4) with a load cell of 50 kN. In the tests, the same sample geometry and boundary conditions were used as for the dynamic tests. A lubricant was also applied to the sample/bar interfaces to reduce friction. Both the DIC technique and displacement transducers were used to measure the deformation of the samples. Three different quasi-static testing speeds of 0.2, 2, and 20 mm/min were imposed to the sample. For both reference quasi-static and high strain rate compression tests, the recorded DIC images were processed using the commercial digital image correlation software MatchID to obtain the local strains after testing. The engineering and true DIC strains were calculated from the developed full displacement field as average strain in an area of approximately 3.5 × 3.5 mm^2^ on the surface of the sample, following the reference Biot and Hencky strain measures, respectively.

## 3. Results and Discussion

The need to use a high T_g_ polymer as toughener for epoxy resin system has driven the choice toward AHBP. The suitable molecular structure consisting of OH– and COOH– substituted benzene rings offers a double advantage: it gives more rigidity and high functionality to improve the cross-linking in filler/matrix system. Furthermore, AHBP can be synthesized by a one-step procedure based on poly-condensation of a unique functional monomer (type AB_x_).

### 3.1. AHBP Characterization

The weight-average molecular weight and molecular weight distribution calculated were 4065 g/mol and 2.7, respectively.

In the HBP structure, trisubstituted, disubstituted, and monosubstituted building blocks can be identified as dendritic (D), linear (L), and terminal (T) unities, respectively. The relative content of them can be used to calculate the degree of branching (DB), which is one of the main structural features for branched polymer, by following equation [32]:

DB = (D + T)/(D + T + L).(3)

The DB value of branched polymer is commonly determined by ^13^C-NMR spectroscopy where D, L, and T are identified by integral intensities of corresponding signals.

The NMR analysis was conducted on both starting monomer and final polymer product. Both samples were dissolved in DMSO-d_6_ which, among the commercially used deuterated solvents, was the only one able to dissolve AHBP.

**Figure 5:**
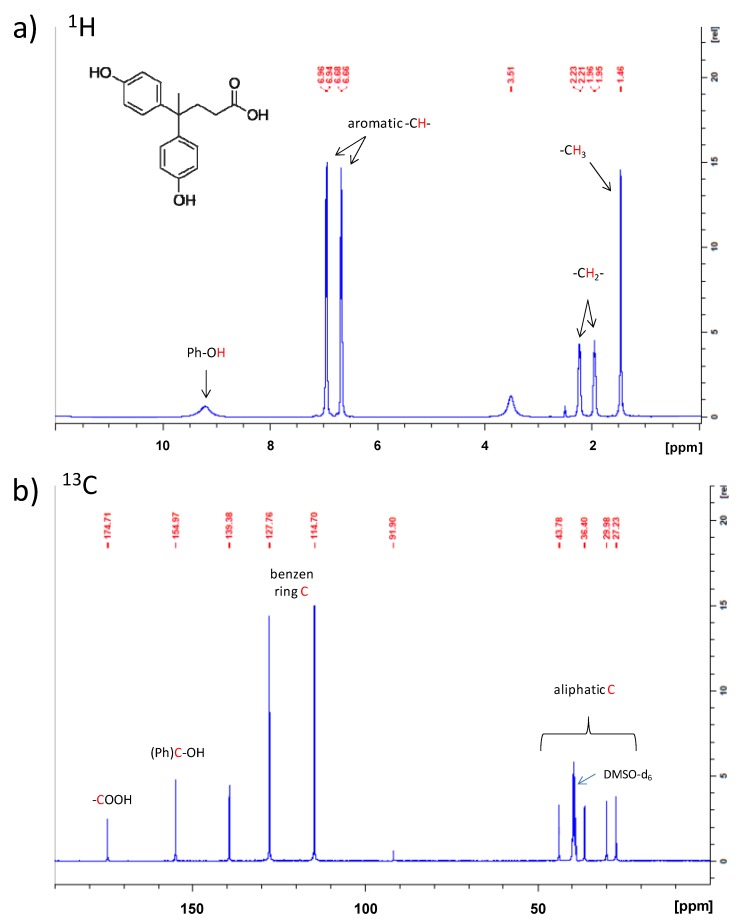
(**a**) ^1^H and (**b**) ^13^C nuclear magnetic resonance (NMR) spectra of diphenolic acid.

The ^1^H-NMR spectrum of diphenolic acid is shown in Figure 5a. The three upfield signals (δ = 1.46, 1.96, and 2.22) are attributable to CH_3_– and –CH_2_– of the aliphatic chain, respectively. The peaks observed at δ = 6.95 and 6.67 are associated with aromatic protons on phenolic rings. Then, at δ = 9.15 a broad, though weak, peak appears, which is attributable to phenolic hydroxyl. Similarly, the ^13^C-NMR spectrum (Figure 5b) shows signals included in the δ = 114–139 range associated with aromatic carbon atoms, and peaks in the δ = 27–45 range related to the aliphatic carbon atoms. The phenolic carbon is indicated by the peak at δ = 154.97, while the carboxylic carbon is clearly identified by signal at δ = 174.71.

**Figure 6:**
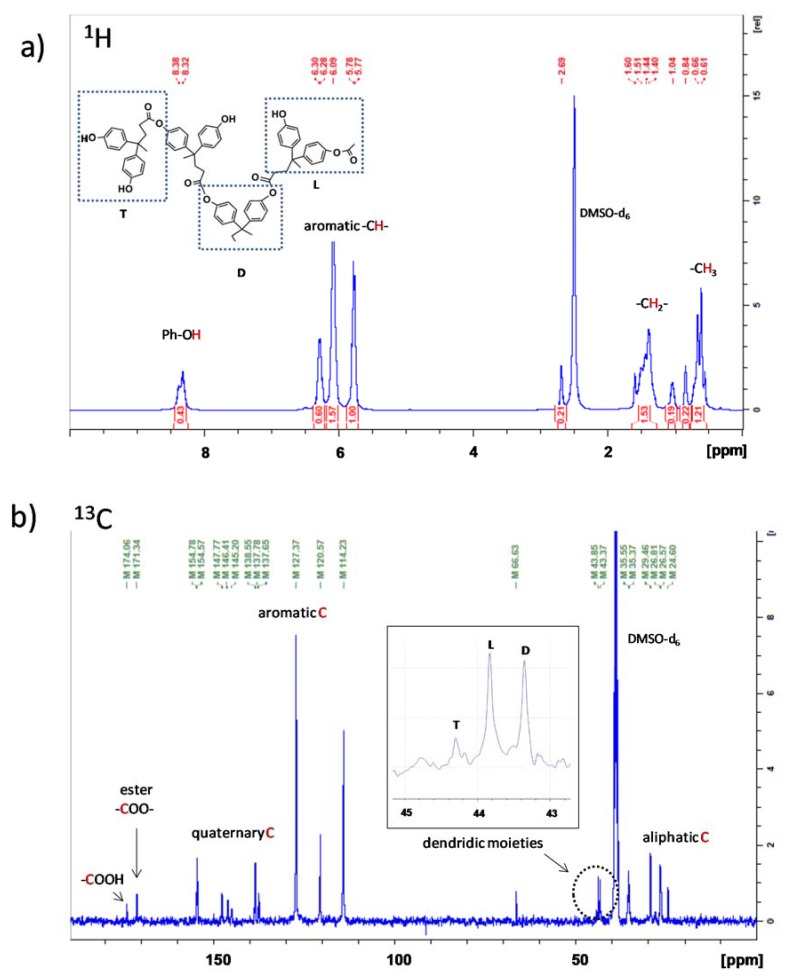
(**a**) ^1^H and (**b**) ^13^C NMR spectra of synthesized AHBP.

The ^1^H-NMR spectrum of the synthesized AHBP is reported in Figure 6a. Despite the complexity of the structure, the main signals are clearly visible. The broad and weak peak associated to the phenolic –OH is still observed but with a shifted value (δ = 8.3) due to the resonance effect of the due to the resonance effect of the formed ester bond with carboxyl groups. A similar behavior is observed for the aromatic and aliphatic protons identified by signals included in δ = 6.8–5.7 and δ = 1.5–0.5 ranges, respectively.

In the ^13^C-NMR spectrum (Figure 6b), no remarkable peaks shifts are observed compared to diphenolic acid. Although, a new peak associated with the ester group appears at δ = 171.34, indicating the polymerization that occurred. Since not all carboxyl groups take part in the esterification reaction, their peaks (δ = 174.06) are still present in the spectrum. Branched unities (T, L, and D) are identified by three signals within the 43–45 of δ-range, whose magnification is reported on the spectrum. Based on these assignments, DB calculated from Equation (3), was 0.57 according to common values for hyperbranched polymers (less than 1) compared to DB (equal to 1) for dendrimers [33].

**Figure 7:**
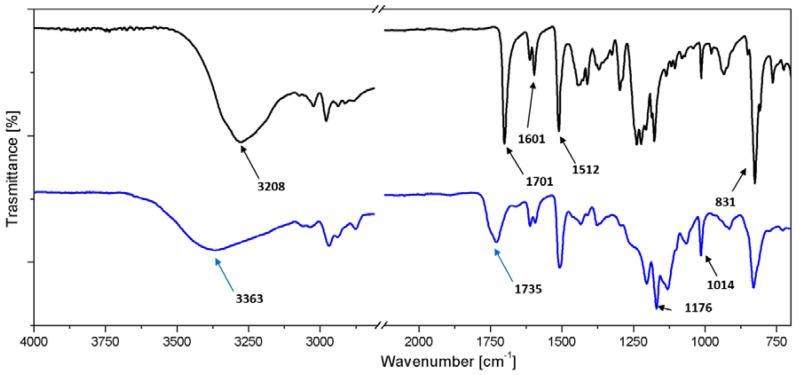
Fourier transform infrared (FTIR) spectra of diphenolic acid (black curve) and AHBP (blue curve).

In Figure 7 the FTIR spectra of diphenolic acid and synthesized AHBP are shown. In the diphenolic acid spectrum (black curve) a broad and strong band is evident, centered at 3208 cm^−1^, associated with the asymmetric –OH stretching vibrations of carboxyl groups and hydroxyl groups. The peak at 1701 cm^−1^ is associated to the C=O stretching vibration, while the peaks at 1601 and 1512 cm^−1^ are both attributable to the C=C stretching vibrations in the benzene rings. In the AHBP spectrum (blue curve), the absence of the peak at 1701 cm^−1^ is noteworthy. It is replaced by a new peak centered at 1735 cm^−1^, associated with the C=O stretching vibration in the ester groups. Peaks at 1176 and 1014 cm^−1^ could be assigned to the asymmetric and symmetric stretching vibrations in the C–O–C bonds, respectively. Para-(1,4)-benzene ring vibrations are associated with the peak at 831 cm^−1^. Since ester groups are formed by the reaction between hydroxyl and carboxyl groups, and consequently some of the hydroxyl groups are removed, the shift from 3208 to 3363 cm^−1^ and the reduction in intensity of the O–H stretching vibration in the AHBP spectrum are in line with expectations.

Table 1 resumes the peaks shown in the FTIR spectra of Figure 7.

**Figure 8:**
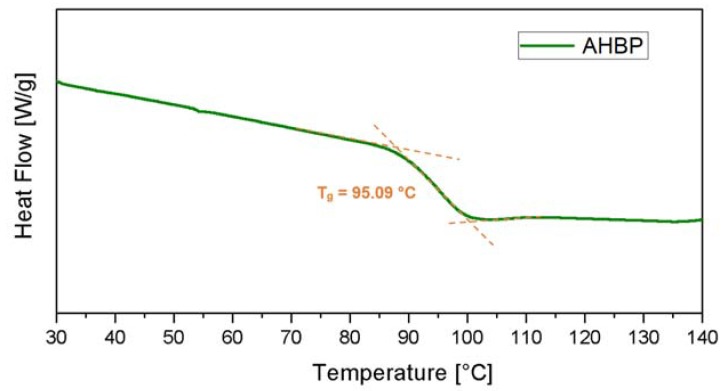
DSC curve of synthesized AHBP.

The synthesized AHBP was also characterized by thermal analysis and a T_g_ value of 95.09 °C (Figure 8) was found. It is well known that the T_g_ depends on the mobility of the macromolecular chain segments: the higher value of the filler T_g_, compared to those found in literature [33,34], is likely attributed to the stiffness of the aromatic segments in the macromolecular chains.

### 3.2. Nanocomposite Characterization

#### 3.2.1. Rheological and Thermal Analysis

**Figure 9:**
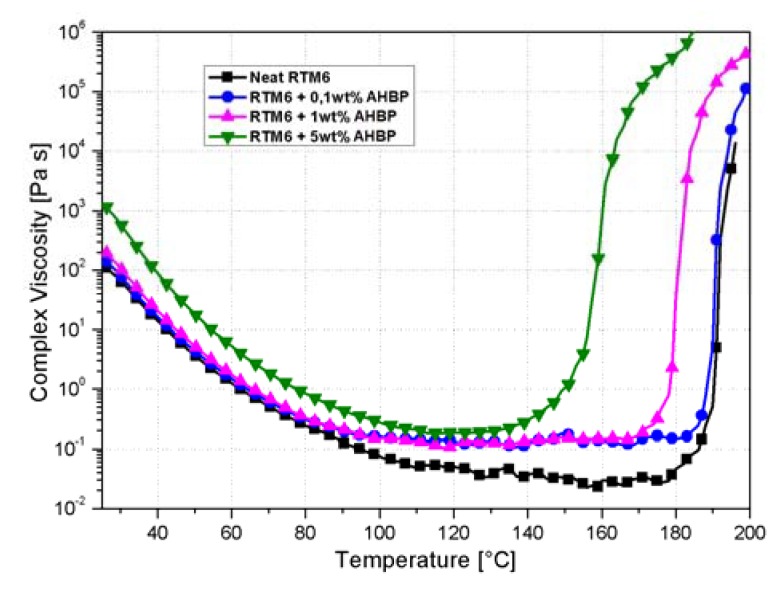
Complex viscosity vs. temperature for the neat system and for suspensions with 0.1, 1, and 5 wt% of AHBP.

Rheological and thermal analyses were performed on uncured neat and filled epoxy systems in order to study the effects of the glassy hyperbranched polymer adducts respectively on viscosity and cure kinetic. Results are reported in Figure 9.

At higher contents, AHBP induces an increase of system viscosity, and this effect is more pronounced in correspondence with the temperatures associated with the minimum viscosity of the hosting matrix (≈120 °C). In particular, the viscosity at 120 °C for the neat resin is about 30 mPa·s, but the addition of 5 wt% of AHBP raises it up to 200 mPa·s, that is about one order of magnitude greater.

In addition to the changes in resin viscosity, Figure 9 also provides useful indications of the catalytic effect of the AHBP for the hosting matrix cure. The presence of the polymeric filler induces a considerable acceleration of the curing process, with a reduction of gelation temperature from 197 to 163 °C. The gelation point can be identified, at fixed heating rate, as the temperature at which viscosity arises above 10^4^ Pa·s. The effect of hyperbranched polyesters on the cure kinetic of an epoxy resin was already studied by Xu et al. [16]: the faster curing process is ascribable to the catalytic effect of hydroxyl groups in the filler and to the low viscosity of the system at curing temperature. Matrix viscosity is one of the main parameters to be considered in several infusions based processes. It directly influences the process filling time and the overall final composite quality. In common vacuum assisted resin infusion molding (VARIM) processes, the matrix viscosity should be lower than 1.0 Pa·s [35].

**Figure 10:**
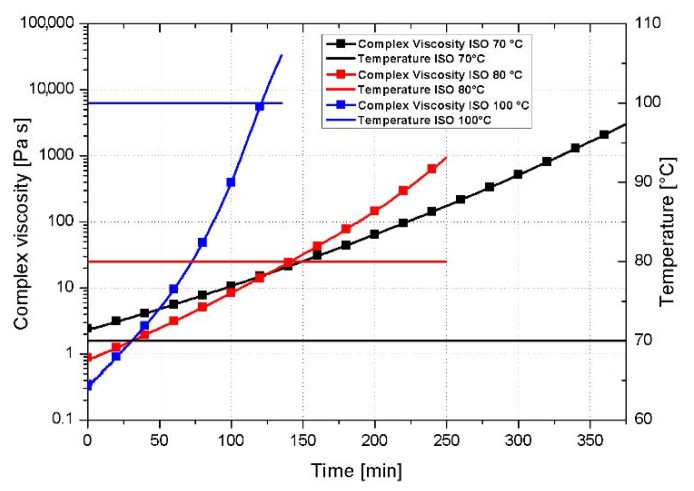
Isothermal rheological tests for the system loaded with 5 wt% of AHBP.

Figure 10 reports the isothermal curves (70, 80, and 100 °C) relative to the system loaded with 5 wt% of AHBP, which reveals the highest value of minimum complex viscosity (≈200 mPa·s at 120 °C). Isothermal rheometric tests at 120 °C are not reported as starting from this temperature the polymerization reaction is almost instantaneously triggered. Obtained viscosity profiles indicate that the system infusion time reduces sharply by increasing the temperature thus limiting the processing time at 100 °C for 25 min, which correspond to the time required to reach 1.0 Pa·s viscosity.

Incorporation of hyperbranched polymers in a matrix can improve the fracture and impact toughness of the resulting epoxy nanocomposites, and likely of the corresponding fiber reinforced composites, but it may affect other mechanical and thermo-mechanical properties, such as the elastic modulus (E) and T_g_, due to the introduction of soft macromolecules into the original matrix network [36]. In order to assess potential variations of both E and T_g_, due to the AHBP loading content, DSC and DMA tests were performed on the manufactured AHBP filled nanocomposites and then compared to the corresponding value of the neat epoxy.

**Figure 11:**
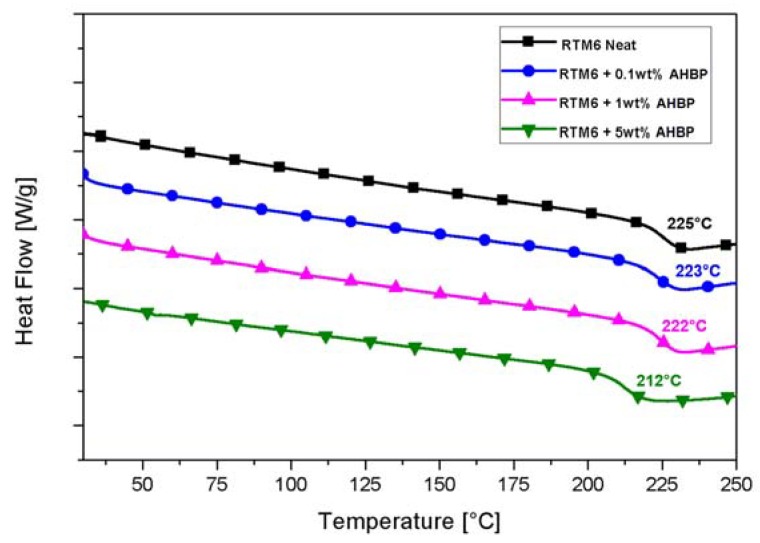
DSC curves of neat and filled systems.

In Figure 11, DSC thermograms of neat RTM6 and AHBP loaded nanocomposites are reported. Similarly to the neat sample, AHBP filled nanocomposites also showed a single glass transition step, indicating the formation of a homogeneous phase due to the good compatibility between filler and hosting matrix.

As it is evident by Figure 11, low AHBP contents (i.e., 0.1 and 1 wt%) induce a negligible change in T_g_, whereas a higher filler content (i.e., 5 wt%), provides a reduction in T_g_ (T_g_ = −12 °C), i.e., from 225 °C for neat RTM6 to 212 °C for the 5 wt% filled samples. The same trend, but with a lower variation, is computed by analyzing the DMA scans and corresponding T_g_ values with an overall change of ≈4 °C for the same systems. In the case of DSC tests, whose main assumption is point-like sample, this reduction is reasonably related to the addition of AHBP phase characterized by its lower T_g_ (≈95 °C), which inevitably will reduce the final system T_g_.

#### 3.2.2. Mechanical Analysis

**Figure 12:**
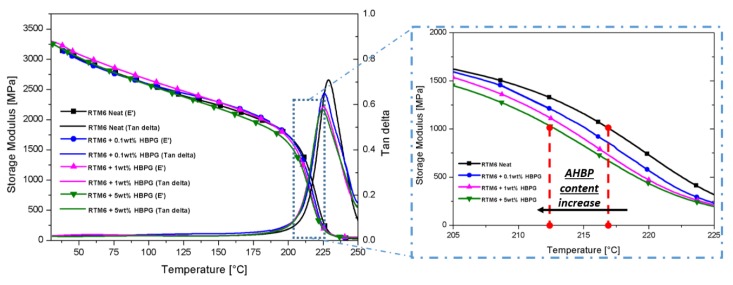
DMA curves of neat and filled systems.

DMA allows the measurement of the dynamic epoxy modulus as function of the temperature (Figure 12). The main results in terms of moduli at 30 °C and at 250 °C, along with the T_g_ for the neat epoxy and AHBP loaded nanocomposites, are reported in Table 2.

Negligible changes of the storage modulus are observable at both temperatures (i.e., 30 and 250 °C) for all filler contents, while a slight reduction of T_g_ is measured for the system filled with the highest content of AHBP (≈4.5 °C). This result, although in line with the DSC data, allows to make two further considerations if analyzed jointly with the trend of storage modulus. In fact, the apparent contradiction associated to the lowering of T_g_ and, at the same time, the increase of glassy and rubbery modulus (up to 1 wt%) could be rationalized by assuming that the level of physical entanglements induced by the presence of AHBP becomes significant at 1 wt% filler content. The effect of both the low T_g_ filler, tending to reduce the overall final system T_g_, and the concentration of entanglements, acting toward a stiffening of the system, would result as balanced at lower filler concentration and maximized at 1 wt%. Furtherly, increasing the weight percentage (i.e., from 1 to 5 wt%), would lead to a coarser level of dispersion and thus a non-uniform density of entanglements weakening the system structure under the applied load. Therefore, the reinforcement efficiency at higher filler content will be lower.

The formed entanglements among the highly rigid AHBP nanoparticles and the hosting matrix chains would affect the level of reinforcement with an optimal content at 1 wt% while at high filler content (i.e., 5 wt%) it will detrimentally affect the reinforcement efficiency.

Fracture toughness tests were performed according to guideline of ASTM D5045 standard. The SENB specimens were subjected to a three-point bending load with a constant cross-head displacement rate of 10 mm/min.

**Figure 13:**
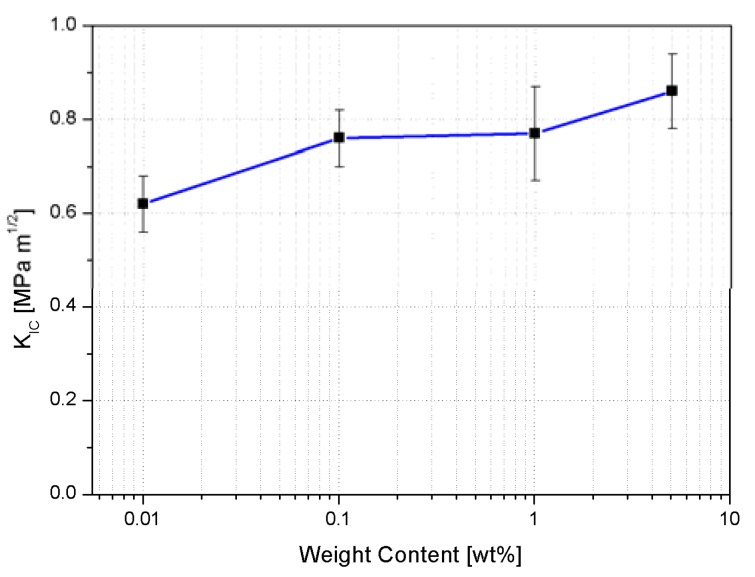
*K_IC_* results obtained from fracture toughness tests for neat and filled systems.

**Figure 14:**
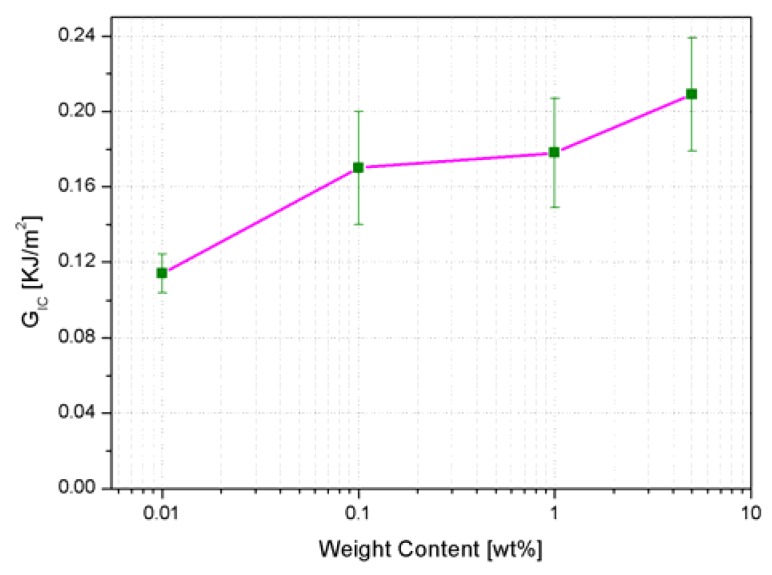
*G_IC_* results obtained from fracture toughness tests for neat and filled systems.

The fracture toughness is substantially expressed by two parameters: *K_IC_*, which is the critical stress amplification factor near the edge of a crack, and *G_IC_*, which represents the energy needed to create two surfaces during a crack propagation. Both the *K_IC_* and *G_IC_* values are remarkably increased by addition of AHBP (Figure 13 and Figure 14). Indeed, it was found that when the content of hyperbranched polymers is only 1 wt%, the *K_IC_* and *G_IC_* of the AHBP/RTM6 system are increased achieving the values of 0.77 MPa m^1/2^ and 0.178 KJ/m^2^, from 0.62 MPa m^1/2^ and 0.113 KJ/m^2^, which represent the corresponding values for the neat resin, respectively.

The highest enhancement of fracture toughness is relative to the samples loaded with 5 wt% of AHBP, characterized by a value of *K_IC_* and *G_IC_* of 0.86 MPa m^1/2^ and 0.209 KJ/m^2^, respectively. The increase of fracture performance is attributable to both a reduction of crosslinking density and a plasticization effect induced by the synthesized filler within the hosting matrix.

**Figure 15:**
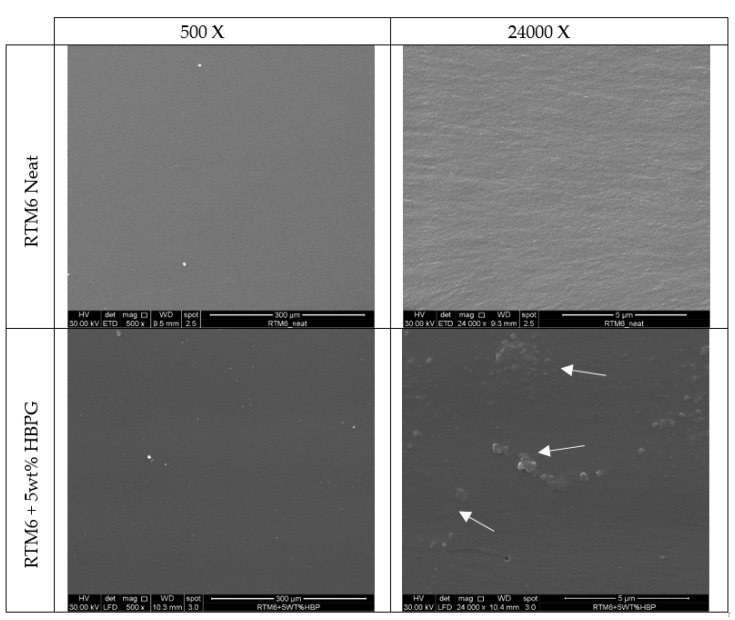
SEM images of fracture surfaces of neat RTM6 and 5 wt% AHBP loaded RTM6 samples.

The mechanism associated with the improvement of the fracture toughness behavior is clearly observable in the SEM images of the fracture surfaces presented in Figure 15. As a matter of fact, the addition of AHBP up to 5 wt% potentially induces plastic deformation of the hosting matrix under a severe deformation state (white arrows in Figure 15). According to this feature, the AHBP/RTM6 system could absorb more energy when cracks occur, justifying the larger increase of critical released energy in forming new surfaces compared to the neat matrix, as shown in Figure 14.

#### 3.2.3. High Strain Rate Test Analysis

**Figure 16:**
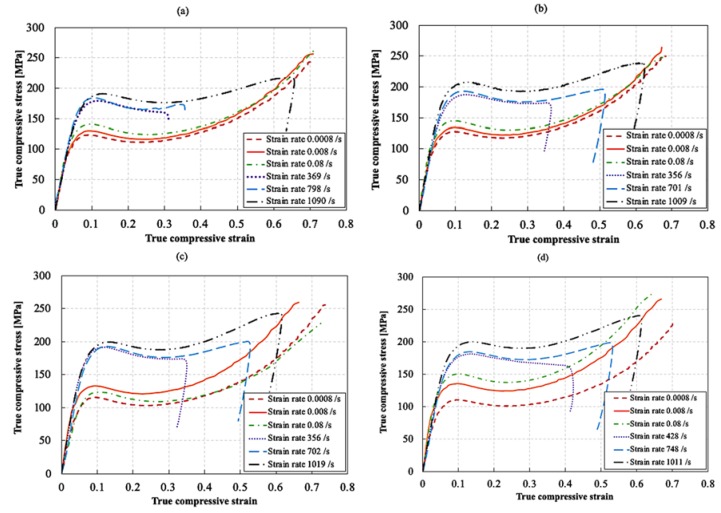
True stress–strain curves at different strain rates for (**a**) neat epoxy, (**b**) 0.1 wt%, (**c**) 1 wt% and (**d**) 5 wt% AHBP epoxy resins.

Figure 16 shows the true stress–strain behavior at different strain rates for the neat and AHBP epoxy resins at different filler concentrations. For each material and test condition, representative curves were selected and presented. At least three experiments were performed for each testing condition. For both the reference quasi-static tests and the high strain rate tests, the true strains obtained from the displacement transducers (corrected for the machine compliance) and from the Hopkinson bar displacement were calculated based on the engineering strain, assuming a conservation of volume.

The true strains obtained from the displacement transducers were in excellent agreement with the strains directly obtained from the DIC up to a strain value of 0.2. Similarly, the strains obtained from the Hopkinson analysis (based on the strain measurements on the bars) also showed excellent agreement with the DIC strains up to 0.2. Moreover, it should be noted that the speckle pattern could not follow the deformation of the samples beyond 0.2 strain in both quasi-static and high strain rate tests. Consequently, the strains higher than 0.2 were based on that obtained from the Hopkinson analysis (for the high strain rate tests) and from the displacement transducers (for the quasi-static tests). The strain showed an initial increase up to a strain level of 0.06, followed by a slight reduction and a relatively constant strain in the range 0.06–0.15. This range was used to calculate the strain rates reported in Figure 16. The strain rates obtained from the tests were in the range of 0.0008–0.08 s^−1^ for the quasi-static tests, and 350–1100 s^−1^ for the high strain rate tests.

The true stress-strain response for all materials can be divided into four main regions: (1) an initial region corresponding to the material’s viscoelastic behavior, (2) a non-linear region corresponding to the yielding of the material, which reaches a maximum value denoted as the peak yield compressive strength, (3) a strain softening region following the yielding, and (4) further strain hardening until failure. For all quasi-static compression tests, the samples were loaded until failure. However, for all the high strain rate tests, the samples did not fail, and a recovery of the strain can be seen upon unloading. The maximum strain achieved in the dynamic tests is limited by the loading duration. Since the samples did not fracture at the end of the tests, the maximum strain reached is not a material property.

It can be seen that the compressive behavior of the neat and AHBP epoxies is strain rate sensitive. All materials show an increase in strength with increasing strain rates. For the AHBP epoxies, also a slight increase in stiffness was observed. Hardly any change in the stiffness was noticed for the neat resin. Both Gerlach et al. [20] and Morelle et al. [37] reported similar trends for RTM6. However, Gerlach showed an increase in stiffness with the increase in strain rate, which seems to contradict the current findings. This could be attributed to the fact that the strain rate range achieved in our study was much lower compared to the range in Gerlach’s work. It should be noted that only for the epoxy with filler content of 1 wt%, a reduction in strength can be seen at a strain rate of 0.08 s^−1^ compared to that of 0.008 s^−1^.

**Figure 17:**
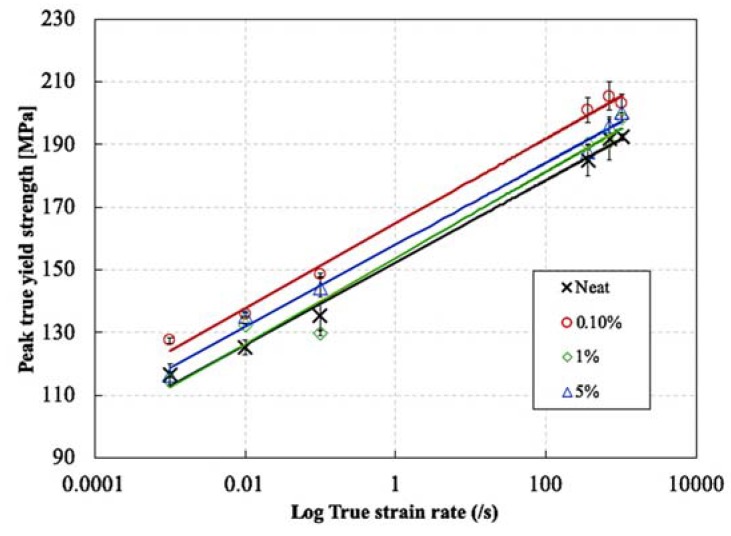
Effect of strain rate on the peak true yield strength neat epoxy resin and different weight content of hyperbranched polymers (HBPs).

Figure 17 shows the effect of strain rate on the true peak yield strength for neat and AHBP loaded epoxy resins with different filler contents. The true peak yield strength was calculated using the DIC strains, assuming a conservation of volume. All tested materials showed an increase in the true peak yield strength at increasing strain rates. Similar results were obtained by Rio et al. [21] for a comparable epoxy resin. It can be seen that the addition of 0.1% and 5 wt% AHBP to the epoxy resin improved the true peak yield strength at different strain rates. The addition of 1 wt% AHBP to the epoxy resin improved the true peak yield strength slightly at high strain rates, while it had almost no effect at low strain rates. Compared to other filler contents, the most significant increase in the true peak yield strength was observed with the addition of 0.1 wt% AHBP. Indeed, at 0.1 wt% filler content, the true peak yield strength increased from 113 MPa for the neat resin to 128 MPa at strain rate of 0.0008 s^−1^, and from 190 to 206 MPa at strain rate of 1000 s^−1^. This corresponds to percentage increases of 13.2% and 8.4%, respectively, compared to the neat resin. On the other hand, a further increase in the AHBP content induced a reduction of the true peak yield strength compared to the sample loaded with 0.1 wt% of AHBP. The true peak yield strength at 5 wt% filler content decreased to approximately 120 MPa at a strain rate of 0.0008 s^−1^ and to approximately 200 MPa at a strain rate of 1000 s^−1^ compared to the respective values of 128 and 206 MPa at 0.1 wt% filler content. However, this still corresponds to percentage increases of about 6.2% and 5.3%, respectively, compared to the neat resin. Furthermore, for the 1 wt% filler content, the true peak yield strength increased to approximately 200 MPa at a strain rate of 1000 s^−1^, which corresponds to an increase of only 4% compared to that of the neat resin. The relative decrease in the true peak yield strength at 1 and 5 wt% AHBP contents compared to the 0.1 wt% AHBP content is attributed to the softening effect associated with the addition of a relatively high AHBP content to the epoxy matrix, as explained earlier. In addition, since the true peak yield strength is realized at a compressive strain of approximately 10%, adiabatic heating effects cannot be neglected, especially at high strain rates [38]. Since the T_g_ of the AHBP is relatively low, i.e., 95 °C, the adiabatic heating could further reduce the strength and stiffness of the AHBPs. This, in turn, might reduce the overall yield strength in the resulting AHBP filled epoxy loaded at higher strain rates.

## 4. Conclusions

The effect of a high T_g_ (95 °C) hyperbranched polymer on thermal, mechanical, and fracture toughness properties of an epoxy resin was investigated.

Three different filler contents were employed (0.1, 1, and 5 wt%), and no remarkable variations of storage modulus, studied through DMA, are reported with the addition of AHBP to the epoxy matrix. DMA also revealed that only the sample loaded with the highest filler content shows a slight reduction of T_g_ (≈4.5 °C). Despite the decrease of T_g_, the fracture toughness is remarkably improved, with an increase of *K_IC_* and *G_IC_* compared to the neat matrix of about 18% and 83%, respectively. According to SEM observations, the improved fracture toughness performance is attributable to a plasticization phenomenon in the hosting matrix induced by the AHBP. Additionally, static and high strain rate compression tests were performed. All materials show a pronounced, positive strain rate sensitivity. The addition of 0.1 wt% of AHBP leads to a significant increase of the peak true yield strength. Indeed, compared to the neat resin, increases of 13.2% and 8.4% are observed at strain rates of 0.0008 and 1000 s^−1^, respectively. Further increasing the filler content lowered the peak true yield strength. Nevertheless, compared to the neat resin, still an overall positive trend in the strength is observed.

## Figures and Tables

**Table 1:** Meaning of FTIR peaks.

Wavenumber (cm^−1^)	Associated With
3366	O–H stretching vibration (hydroxyl and carboxyl groups)
1728	C=O stretching vibration (ester group)
1611, 1509	C=C stretching vibration (benzene ring)
1169, 1014	C–O–C asymmetric and symmetric vibration (ester group)
831	Para(1,4)-benzene ring vibration

**Table 2:** Neat and filled systems DSC and DMA results.

Sample ID	T_g_^DSC^ (°C)	T_g_^DMA^ (°C)	E’ (30 °C)	E’ (250 °C)
RTM6 Neat	225.1	228.9	3246 ± 34	40 ± 2.5
RTM6 + 0.1 wt% AHBP	223.4	225.6	3261 ± 29	41 ± 3.7
RTM6 + 1 wt% AHBP	222.2	225.2	3340 ± 49	47 ± 5.0
RTM6 + 5 wt% AHBP	212.9	224.4	3263 ± 37	42 ± 2.1
